# Enhanced Mechanical and Durability Properties of Cement Mortar by Using Alumina Nanocoating on Carbon Nanofibers

**DOI:** 10.3390/ma15082768

**Published:** 2022-04-09

**Authors:** Huda Al Qader, Ahmed M. Jasim, Hani Salim, Yangchuan Xing, David Stalla

**Affiliations:** 1 Department of Environmental and Civil Engineering, University of Missouri, Columbia, MO 65211, USA; noorih@mail.missouri.edu; 2 Department of Biomedical, Biological, and Chemical Engineering, University of Missouri, Columbia, MO 65211, USA; amjtg6@mail.missouri.edu (A.M.J.); xingy@missouri.edu (Y.X.); 3 Electron Microscopy Core Center, University of Missouri, Columbia, MO 65211, USA; ds5x8@missouri.edu

**Keywords:** carbon nanofibers, nanocoating, alumina, durability

## Abstract

This study evaluated the effect of carbon nanofibers (CNFs) coated by aluminum oxide Al_2_O_3_ as a reinforcement on compressive strength, frost resistance, and drying shrinkage of cement mortars. Three weight ratios of 0.125%, 0.25%, and 0.5% of Al_2_O_3_/CNFs and bare CNF cement mortars were compared with reference cement mortar samples. The reactive porous and high surface area layer of alumina induced the hydration reaction and promoted the production of well-distributed hydration gel. Derivative thermal analysis–differential thermogravimetric (TGA-DTG) and X-ray powder diffraction (XRD) characterization showed that Al_2_O_3_/CNFs reinforcement led to greater hydration gel production than bare CNFs. Transmission electron microscopy (TEM) and scanning electron microscopy (SEM) were performed to study the coating and microstructure of the cement mortars evaluated in this paper. The results show that the optimum enhancement of the cement mortar properties was obtained at ratios of 0.125% for Al_2_O_3_/CNFs and 0.25% for CNFs. This enhancement was greater with Al_2_O_3_/CNFs-reinforced specimens in terms of high compressive strength, less compressive strength degradation after 150 cycles, and less drying shrinkage. The low use of the CNFs in Al_2_O_3_/CNFs samples indicates the coating is an economical and promising approach for improving the performance of cement mortars.

## 1. Introduction

Cementitious materials are the most prevalent construction materials nowadays, but they suffer from low tensile strength and are highly susceptible to cracking. Therefore, several research efforts have been devoted to enhancing the cement structure with macro-level or micro-level reinforcements [1,2,3,4] However, internal voids and cracks generally start from a nano size as embryos, then grow to a micro size, leading to a destructive level. If the voids’ population is largely present in the cementitious structure, the water easily penetrates and precipitates them, which can lead to freeze–thaw damage [5]. Exposing the cementitious materials to repetitive freeze–thaw cycles severely deteriorates their structure. Therefore, nano-scale reinforcements are used to prevent voids’ initiation as an effective way to protect the inherent mechanical and durability properties [6,7,8,9]. Researchers have found that nanomaterials are highly reactive to cementitious materials as they can act as seeding sites for the hydration products [10,11]. The hydration reaction is catalyzed by either oxygen functional groups (-OH, -COOH, -C=O) on carbon nanomaterials or the pozzolanic reactivity associated with some metal oxide nanoparticles (SiO_2_, Al_2_O_3_) [12]. This mainly serves for porosity refinement, water impermeability, and structure bridging.

Recently, there has been a growing interest in integrating carbon nanomaterials, such as carbon nanotubes (CNTs), carbon nanofibers (CNFs), and graphene [13,14,15], in cement composites to increase their frost resistance. The efforts have focused on either eliminating the void formation or refining them to small sizes to reduce the permeability of the cementitious material, hence improving the durability. Others have reported allowing extra space for ice to expand by incorporating air bubbles in the composites [12]. Limited studies have explicitly reported on using CNFs in enhancing the cement frost resistance. Cwirzen et al. [16] studied the effect of the CNFs on the strength and residual cumulative strain of cement mortars after freeze–thaw cycles with a high water-to-cement ratio of 0.5. They found that the higher the content of the CNF composites, the lower the ultimate strain and the internal damage. Their study showed the bridging ability of CNFs with higher water ratios. Wang et al. [9] comparatively investigated the different CNFs’ loading effect on frost resistance. They found a 0.3% CNF dosage to be an optimum choice that refined the microstructures, which promoted the durability. Metaxa et al. [17] demonstrated the ability of the CNFs to arrest crack growth in the cement matrix. According to their results, the CNFs performed well in reducing the crack propagation by a bridging phenomenon that provided a load transfer mechanism across cracks and pores. However, the above-mentioned properties of the addition of the CNFs to the cementitious materials are limited by the dispersion of CNFs [18] and by the bonding with the cement matrix. Therefore, surface treatment is essential to improve the solubility as well as the bonding with cement hydration products.

Metal oxide nanoparticles (MNPs) have been vastly utilized in reducing the porosity of cement composites. These materials possess a high surface area that makes them reactive, strengthening the microstructure of the cement with less porosity [19,20], especially those that possess a pozzolanic property, such as silica and alumina nanoparticles [20,21,22,23]. Those materials react with calcium hydroxide Ca (OH)_2_, which has portlandite, forming a secondary reaction producing gels of calcium–silicate–hydrate (C-S-H) and calcium–aluminum–hydrate (C-A-H). The rate of such a reaction is controlled by the available surface area and the surface nucleation sites. Behfarnia et al. [21] discussed the feasibility of using both SiO_2_ and Al_2_O_3_ nanoparticles in enhancing the frost resistance of the concrete mixes due to the pozzolanic and refining effects. Muzenski et al. [24] reported that using 0.25 wt.% of aluminum oxide nanofibers increased the compressive strength by 30%.

However, an inhomogeneous dispersion of these nanoparticles could devolve their functions, such as forming aggregates and inducing formation of large-size voids. Unlike CNFs or CNTs, MNPs lack a bridging phenomenon, which is highly needed to compact the cement microstructure. Another issue with MNPs is their cost, which prohibits it from being scaled up. Therefore, an innovative design for metal oxide nanoparticles with a high surface area, hydrophilicity, and low cost is needed.

Drying shrinkage is another property the community has sought to practically solve. Different nanomaterials have been applied, aiming to reduce shrinkage. The aforementioned nanomaterials have also been used to improve drying shrinkage response resulting from many effects, such as surface hydrophilicity of CNF [25], fast hydration reaction of SiO_2_ [26], and formation of the secondary moieties of C-S-H and C-A-H [27].

Limited research exists on combining carbon nanomaterials and metal oxides as a promising approach in enhancing the mechanical properties and the durability of cement through improving the interfacial interaction of the carbon materials with cement, enhancing the dispersion and retaining the bridging [28,29,30,31,32,33,34]. Such combinations are achieved by incorporating nanoparticles or metal oxide nanocoating. Sanchez et al. [28] used silicon powder with CNFs to improve the dispersion of CNFs and promote the interfacial interaction between the cement paste and the CNFs. Kim et al. [33] reported an improvement in the CNTs’ dispersion and in enhancing the cement mechanical properties by incorporating silica fumes with CNTs. Garg and Das [32] showed a significant improvement in carbon nanotubes’ dispersion and bonding due to a pozzolanic reaction after silica nanoparticles’ incorporation. Sikora et al. [31] has reported a comparison between CNTs and CNT/NS on the compressive strength of cement pastes under elevated temperatures. They found that adding silica in the form of a nanocoating with a dense layer has a favorable effect on increasing the bond and protecting the CNTs from being oxidized at high temperatures. Dong et al. [34] have reported a study on coating the CNTs with nickel nanoparticles and their role in the concrete. They studied different aspect ratios and volume loadings in the cement microstructure. They found coupling Ni/CNTs increased the dispersion and the compressive and flexural strength.

To our knowledge, no study has reported using aluminum oxide as a coating layer to improve mechanical properties and durability of cement mortars. Therefore, this paper reports on a core–shell structure of carbon nanofibers (CNFs) as the core and a porous, fluffy alumina Al_2_O_3_ as the shell, denoted Al_2_O_3_/CNFs, at a by-weight loading of 42% and 58% of Al_2_O_3_ and CNFs, respectively. The advantages of using such materials are a low cost, high specific surface area, high dispersion with pozzolanic property, and a bridging property. This study investigated the compressive strength, the freeze–thaw resistance, and drying shrinkage of the cement mortar. Three ratios of 0.125%, 0.25%, and 0.5% of Al_2_O_3_/CNFs and bare CNFs were compared with a standard sample. Our results show the 0.125% Al_2_O_3_/CNFs and 0.25 wt.% CNFs performed the best out of all tests. We deduce the reason for such a performance is that the Al_2_O_3_ nanolayer accelerated the production of the hydration gel. The gel production was confirmed by the thermal analysis (TGA) and XRD results.

## 2. Experimental Program

### 2.1. Materials

Ordinary Portland cement classified within the ASTM C150 standard type I was utilized to prepare the mortar mix. Natural river sand with a fineness modulus of 2.6 was used in all mixes. To enhance the workability, a water-reducing admixture was used. CNFs with a diameter ranging between 50 and 150 nm were used in this study. For coating, trimethylaluminum (TMA-1.0M) was used as a precursor.

### 2.2. Synthesis of Al_2_O_3_ Coating on CNFs

The synthesis of Al_2_O_3_ coating on CNFs was conducted by using a condensed layer deposition (CLD) [35]. The CNFs were surface-treated by a mixture of sulfuric acid and nitric acid at a volume ratio of 3:1 at a concentration of 6.0 M under a sonication probe for 2 h at 60 °C followed by deionized water washing to remove any acid residue. The coating process was prepared in batches where each batch of 400 mg of CNF was dispersed in 500 mL of heptane in two containers, 200 mg of CNFs in 250 mL each. After a 20 min period of sonication, 150 microliters of water were added to the mixture under both sonication and stirring. After another 20 min, a water film formed on the CNFs surface. Then, a 0.00789 mole of the TMA based on the reaction 2TMA + 3H_2_O → Al_2_O_3_ + 6CH_4_ was injected under a nitrogen blanket to prevent any atmosphere effect. The samples were dried under vacuum at 100 °C for 8 h. Subsequent annealing to remove any residues in the form of CHx was conducted at 350 °C for 2 h under air in a tubular furnace.

### 2.3. Fabrication of Mortars

**Figure 1:**
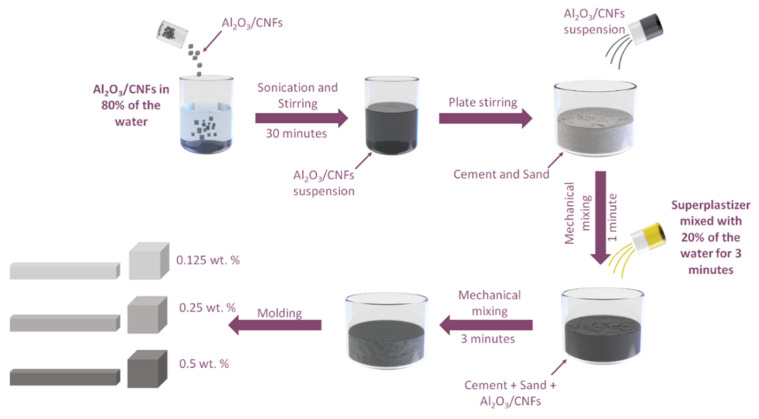
Schematic of preparation process.

A constant cement-to-sand ratio of 1:2 and a water-to-cement ratio of 0.35 were used. Three different ratios of CNFs and Al_2_O_3_/CNFs at weights of 0.125%, 0.25%, and 0.5% were prepared. In addition, a control specimen without nanomaterial, denoted as C0, was prepared (mixing properties are listed in Table 1). In a typical procedure, CNFs and Al_2_O_3_/CNFs suspensions can be prepared by sonicating them (at 800 W–20 kHz) in water while the mechanical stirring is ongoing for 30 min. This water can then be used as a mixing agent to prepare the composite mortars. To achieve fluidity, a certain ratio of a superplasticizer was added (see Table 1) to the mixture of water and nanomaterials or to water only. The superplasticizer, as a surfactant, was used only with the suspensions that had only CNFs. In the case of Al_2_O_3_/CNFs, 80% of water was used for Al_2_O_3_/CNF dispersion and 20% was mixed with superplasticizer. Figure 1 illustrates the mixing procedures for Al_2_O_3_/CNFs composites. Afterward, the fresh mixture was cast into different molds for the different testing procedures described in this paper.

### 2.4. Characterization Techniques

#### 2.4.1. Surface Characterizations

Two types of surface characterizations were adopted. Transmission electron microscopy (TEM) was used to evaluate the Al_2_O_3_ coating on CNFs using Jeol-1400 at an acceleration voltage of 120 kV. Scanning electron microscopy (SEM) FEI Quanta 600 FEG SEM scope was used to investigate the cement internal structure. The SEM scope voltage was set at 5 kV to provide superior sensitivity to image surface structure. The surface area, whether that of Al_2_O_3_/CNFs or that of the cement composites, was measured using the Brunauer–Emmett–Teller method (BET).

#### 2.4.2. Thermogravimetric Analysis

To exactly measure the Al_2_O_3_ coating layer loading on the CNFs and the sample overall, thermal analysis (TGA) model Q-500 (TA Instruments, Newcastle, DE, USA) was conducted in a range of 25–1000 °C with a scan rate of 10 °C/min. TGA-DTA was also performed to measure the Ca (OH)_2_ content of the control and CNFs-0.25 and Al_2_O_3_/CNFs-0.125 samples at the ages of 7, 14, and 28 days. No special treatment, such as drying or pouring in any solvent, was used. Cement mortar slices of 5–8 mm were taken from the core of the sample cubes after being tested under compression to failure. The slices were crushed to powders and filtered by passing them through a 75 µm sieve. In each test, about 30 mg of sample was heated under nitrogen gas at a range of 25–1000 °C at a heating rate of 10 °C/min.

#### 2.4.3. X-ray Diffraction Analysis

X-ray diffraction (XRD) (Philips X-Pert-Fisher Scientific, Hampton, NH, USA) equipped with CuKα was performed to analyze the crystalline phase of the samples. The data were collected for an angle range of 2θ = 5°–90° at a scan rate of 0.026° s^−1^. The samples (50 mg in weight) were grained and sieved with a 75 μm sieve.

#### 2.4.4. Fluidity and Compressive Strength Tests

To evaluate the effect of the addition of Al_2_O_3_/CNFs and CNFs on the cement mortars, fluidity and workability of different composite mixtures were examined based on ASTM C1437-13 standards [36]. After the mixing procedure was carried out, a specific amount of each mixture was poured into the flow table following ASTM standards. The compressive strength after 7, 14, and 28 days was determined following the procedure of the ASTM C109 standards [37]. Sixty-three specimens with dimensions of 50 mm × 50 mm × 50 mm were tested. The specimens were removed from the lime water solution and were wiped dry 24 h before testing.

#### 2.4.5. Freeze–Thaw Tests

To study the durability of the bare and reinforced mortars, freeze–thaw cycling process was conducted at a temperature range of −18 °C to 4 °C based on ASTMC666, rapid freeze–thaw cycling in water [38]. The specimens were prisms of 25 mm × 25 mm × 280 mm dimensions. Before the freeze–thaw test, the specimens were demolded and moist-cured for 28 days. The samples were subjected to at least 300 freeze–thaw cycles. The loss of mass of all composite mortars and the reduction in compressive strength of composites with optimum dosage of nanomaterials was measured after a specified number of freeze–thaw cycles.

#### 2.4.6. Drying Shrinkage Tests

Cement mortar samples with w/c ratio of 0.4 with dimensions of 25 mm × 25 mm × 280 mm were used for drying shrinkage measurements in accordance with ASTM C596 [39]. Samples were demolded after 48 h after casting and placed in lime water. After 72 h had passed since casting, the specimens were removed from the lime water and the initial dial gauge measurement of the length were recorded. The length measurements were conducted using length comparator according to the ASTM C490/C490M-17 [40]. The specimens were stored in a dry room at 22 ± 4 °C and 90% relative humidity. The length change measurements were taken at the ages of 5, 7, 14, 28, and 56 days.

## 3. Results and Discussion

**Figure 2:**
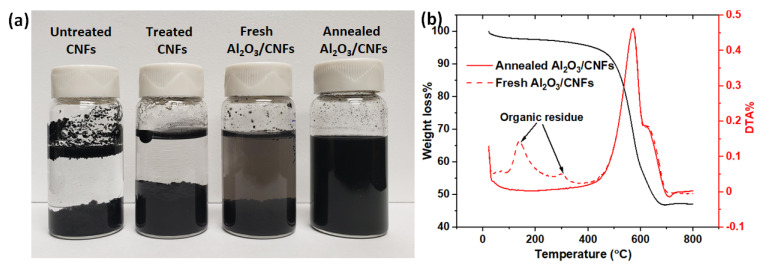
(**a**) CNF and Al_2_O_3_/CNF dispersion in water after one month, and (**b**) TGA-DTA profile for Al_2_O_3_/CNFs before and after annealing at 350 °C under air.

Dispersion of bare CNFs and Al_2_O_3_/CNFs dispersed in water before and after surface functionalization is shown in Figure 2a, and Al_2_O_3_/CNFs before and after the annealing at 350 °C under air is shown in Figure 2b. The observations were made after the 30 min sonicating process without the addition of any surfactant, and the different solutions were left for 30 days. It could be seen that the untreated CNF solution showed no dispersion, while the treated CNFs solution showed relatively good dispersion. However, after 30 days, some CNF clusters were clearly visible at the bottom of the vials. The fresh Al_2_O_3_/CNFs settled down while in water, which was due to the organic ligands left over from the coating precursor molecule. The scenario was totally different after the fresh Al_2_O_3_/CNFs were exposed to air at a temperature of 350 °C for one hour. Therefore, annealed Al_2_O_3_/CNFs suspensions had much better long-term stability as compared with other suspensions. The removal of organic leftovers was confirmed by the TGA analysis, as shown in Figure 2b. The better dispersion is attributed to the formation of pores due to oxidizing the organic content, converting them into highly hydrophilic surfaces.

The findings of this paper provide fundamental evidence that could lead to practical implications in the future. The methodology and results obtained at a small scale can be advanced further with additional research to potentially impact current engineering practice. Specific discussion is provided next for the various results obtained in this paper.

### 3.1. Transmission Electron Microscopy (TEM)

**Figure 3:**
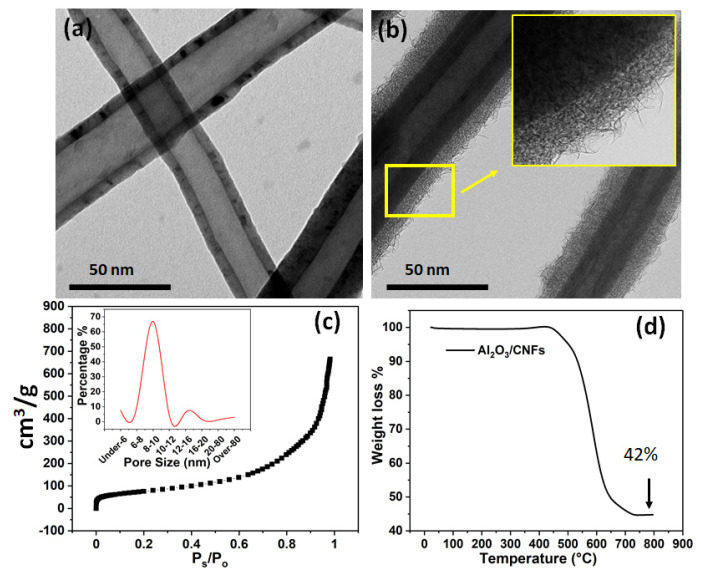
Bright-field transmission electron microscopy (TEM): (**a**) uncoated CNFs, (**b**) Al_2_O_3_ coating on CNFs showing a close-up of the layer morphology, (**c**) BET analysis for Al_2_O_3_/CNFs with its pore size distribution, and (**d**) TGA analysis showing mass loading of the Al_2_O_3_ on CNFs.

The nanocoating of Al_2_O_3_ on CNFs, as characterized by TEM at both low and high resolution, is shown in Figure 3. Bare CNFs were examined by TEM as well, as shown in Figure 3a. Figure 3b shows a uniform coating with a porous morphology, which plays a role in the cement hydration. TGA analysis showed 42% and 58% weight percent for Al_2_O_3_ and CNFs, respectively. Figure 3c displays the adsorption scan of the nitrogen gas for surface area measurement. The surface area was found to be 274.36 m^2^/g as well as the nanocoating layer pore size distribution. Figure 3d shows the TGA analysis for the mass loading of Al_2_O_3_ on CNFs, which was found to be 42 wt.%.

### 3.2. Microstructure Analysis

**Figure 4:**
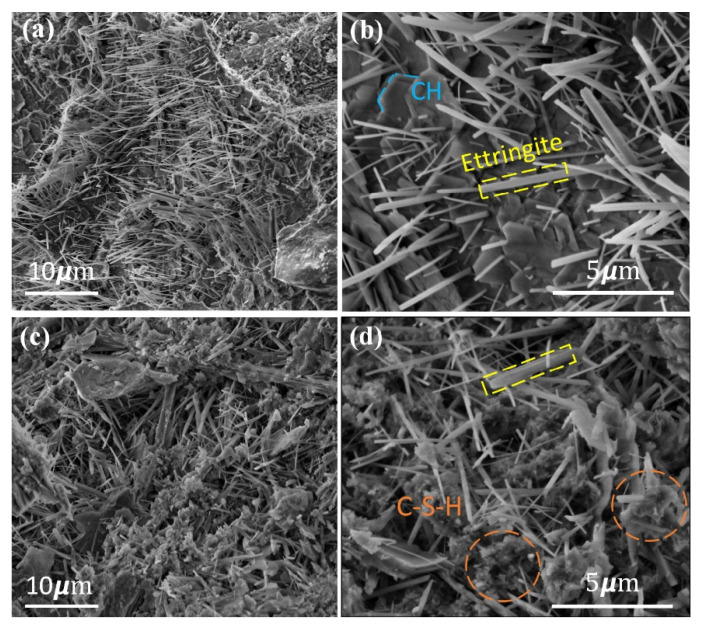
SEM images at different magnifications (**a**,**b**), CNFs embedded in cement mortar, and (**c**,**d**) Al_2_O_3_/CNFs embedded in cement mortar, all at the age of 28 days.

The microstructure of the cement mortar specimens was characterized by scanning electron microscopy (SEM). Figure 4 shows the SEM images for CNFs and Al_2_O_3_/CNFs at the age of 28 days, where the hydration products such as crystalline CH, needle-shaped ettringite, and amorphous calcium–silicate–hydrate (C-S-H) are clearly distinguished. The SEM session was performed at a voltage of 5 kV to avoid any surface charging under the beam exposure. Figure 4 shows the CNFs and Al_2_O_3_/CNF-embedded cement mortar (marked by arrows). Al_2_O_3_/CNFs was found to be well-anchored in the hydration products and was encapsulated by uniform and compacted C-S-H hydration products. The increased C-S-H gel formation is attributed to the contribution of the Al_2_O_3_ nanocoating as an effective layer with a high surface area that provides preferential nucleation sites for hydration products’ growth. In addition, based on the observation of the surface morphology, a lower CH content was found when compared with CNFs-mortar composites. This could be due to the consumption of CH formed during the hydration of the cement [21]. On the other hand, the fracture surface for the CNFs composites showed a good interaction with the mortar matrix with a high CH content.

It is predicted that the porous morphology of Al_2_O_3_ enhances the hydration reaction as the porous sites adsorb the water, which becomes a nucleation site for the cement. The ratio of Ca/Si was used as a metric to determine the consumption of calcium converted to a hydrated silicate moiety [41]. The EDS analysis found the Ca/Si ratio was the highest in the case of C0 specimens, whereas it had the lowest value for Al_2_O_3_/CNFs-0.125% composite specimens, which further supports the findings from TGA and XRD data [42].

### 3.3. Cement Hydration by Thermal Analysis

Thermogravimetric (TGA) and differential thermogravimetric (DTG) curves were obtained to detect the influence of CNFs and Al_2_O_3_/CNFs in the hydration reaction of cement mortars at a water/cement ratio (w/c) of 0.35. In general, the decomposition of cement hydrates can be divided into three major regions. The first peak between 25 and 400 °C represents the evaporable water and dehydration of hydrated calcium silicate (C-S-H). The second one between 400 and 600 °C represents the dihydroxylation of portlandite (CH). As the CNFs started breaking down in this temperature window [43], this could be attributed to some overlapping between the CH and CNFs’ thermal oxidation signal. The last peak due to decarbonation of CaCO_3_ is between 600 and 800 °C [44].

**Figure 5:**
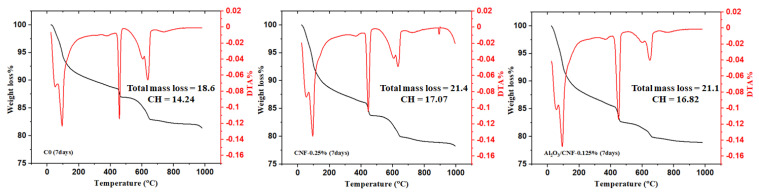
TGA analysis for C0 (**left**), CNFs-0.25 (**middle**), and Al_2_O_3_/CNFs-0.125 (**right**) at a curing age of 7 days.

**Figure 6:**
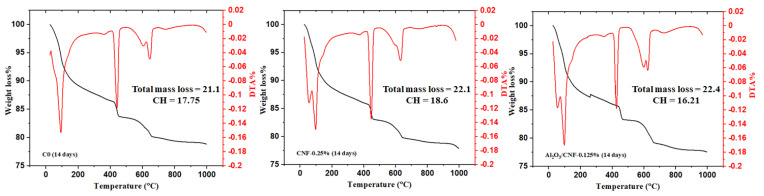
TGA analysis for C0 (**left**), CNFs-0.25 (**middle**), and Al_2_O_3_/CNFs-0.125 (**right**) at a curing age of 14 days.

**Figure 7:**
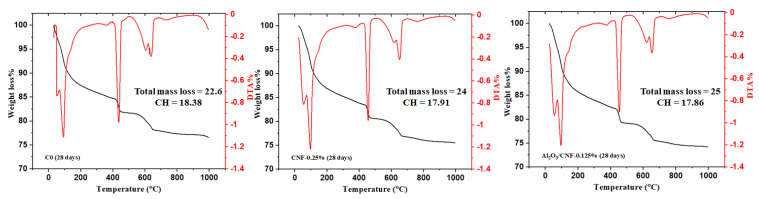
TGA analysis for C0 (**left**), CNFs-0.25 (**middle**), and Al_2_O_3_/CNFs-0.125 (**right**) at a curing age of 28 days.

The TGA and DTA results for the cement mortar composites for the standard C0, CNFs-0.25%, and Al_2_O_3_/CNFs-0.125% were collected at the ages of 7, 14 and 28 days, as shown in Figure 5, Figure 6 and Figure 7, respectively. Compared with C0, the samples with nanomaterials showed an increase in the total mass loss. This is an indicator that the addition of the nanomaterial promoted the degree of hydration, but the Al_2_O_3_/CNF nanomaterials showed more significant effects at 14 and 28 days of curing age. One of the important metrics in assessing the extent of the hydration progress is determining the CH content. At 7 days, the largest amount of CH was observed in CNFs-0.25% and Al_2_O_3_/CNFs-0.125% at 17.1 and 16.82%, respectively. When increasing the curing age to 14 days, the CH content in C0 and CNFs-0.25% reached 17.75% and 18.6%, indicating that the degree of hydration increases with age. However, the CH content in the Al_2_O_3_/CNFs-0.125% composite was the lowest among the samples. This could also be explained due by the reactivity of the porous layer of alumina, which functions as an effective seeding site for hydration products’ increase in CH and C-S-H. More uniform C-S-H can densify and compact the mortar matrix and restrict the growth of the crystal CH. The content of CH at the age of 28 days was also reduced in the case of CNF composites. Based on these results, one can conclude that the addition of CNFs and Al_2_O_3_/CNFs increases the degree of hydration by providing preferential nucleation sites at its oxygen functional groups. The effect of the covalent bonding provided by surface functional groups on CNFs or by the porous alumina govern its performance [45].

### 3.4. X-ray Diffraction (XRD)

**Figure 8:**
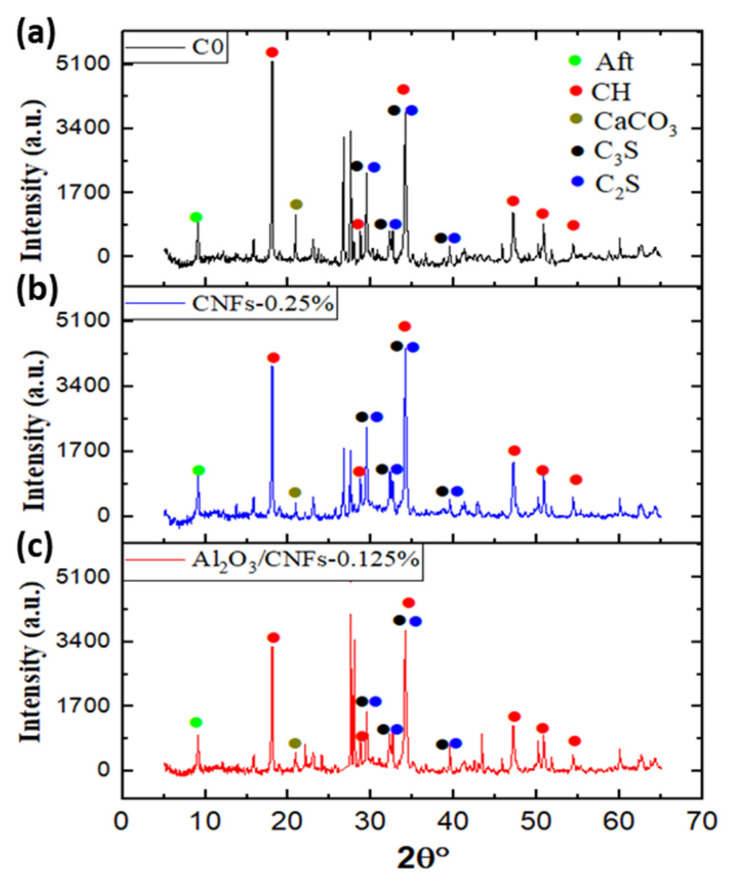
XRD patterns’ total spectrum: (**a**) C0, (**b**) CNFs-0.25, and (**c**) Al_2_O_3_/CNFs-0.125%.

To qualitatively investigate the mineralogical compositions for the mortar composites, XRD patterns were performed. The results for samples C0, CNFs-0.25%, and Al_2_O_3_/CNFs-0.125% at the age of 28 days are displayed in Figure 8. It can be seen that the XRD patterns for all samples show identical diffraction angles. The phases found in all mixtures were ettringite (Aft), portlandite (CH), tricalcium silicate (C_3_S), dicalcium silicate (C_2_S), and calcium carbonate CaCO_3_. The diffraction angle of 2θ = 9.1° belongs to ettringite, whereas the peak at 2θ = 22.5° is for CaCO_3_. The peaks at 2θ = 18°, 28°, 47°, 51°, and 54° are attributed to calcium hydroxide (CH). The diffraction peaks at 2θ = 29.5°, 32°, 34°, and 39.3° were assigned to the anhydrate C_3_S and C_2_S [46]. The cement clinker was composed of about 75% chemical components of C_3_S and C_2_S. These two components react with water to produce crystalline calcium hydroxide or portlandite (CH) and the amorphous form of calcium–silicate–hydrate (C-S-H). In addition, these two hydration products comprise over 60% of hydration products. The chemical formulas expressing the hydration reaction of C_3_S and C_2_S are represented by the equations below:

C_3_S + H_2_O → C-S-H + CH

C_2_S + H_2_O → C-S-H + CH

Therefore, measuring the peak intensities of C_2_S and C_3_S gave us a sense of how much C_2_S and C_3_S had been consumed during the hydration reaction. The intensity of the peaks of C_3_S and C_2_S at 2θ = 29.5° was higher in the control sample. The peak intensity decreased when CNFs-0.25% was embedded, and a significant reduction in the peak was observed when Al_2_O_3_/CNFs-0.125% was in the cement matrix. The results indicate that hydration proceeds more efficiently in Al_2_O_3_/CNFs followed by CNFs-0.25% and finally C0. Another noted observation from the XRD data is the CH peak at 2θ = 18°, as shown in Figure 8. It was observed that the peak decreased in the case of Al_2_O_3_/CNFs-0.125% relative to the other two samples. This indicates the influence on the mortar hydration by accelerating the dissolution of C_3_S and C_2_S, leading to an increase in and better distribution of gel hydration C-S-H. This growth may restrict the growth of CH, as can be observed in the SEM morphology images (Figure 4d). The lesser CH intensity may also be an indicator of formation of an additional C-A-H gel [21,22,23,47]. The hydration gels are amorphous in nature and are not detectable in XRD. Therefore, an indirect measurement can be adapted by focusing on the CH intensity and anhydrate C_3_S and C_2_S phases. In conclusion, the addition of the CNFs and Al_2_O_3_/CNFs determined the degree of the hydration, and this promotion was significant in the case of Al_2_O_3_’s presence. These results are in line with the TGA findings described earlier.

### 3.5. Compressive Strength

**Figure 9:**
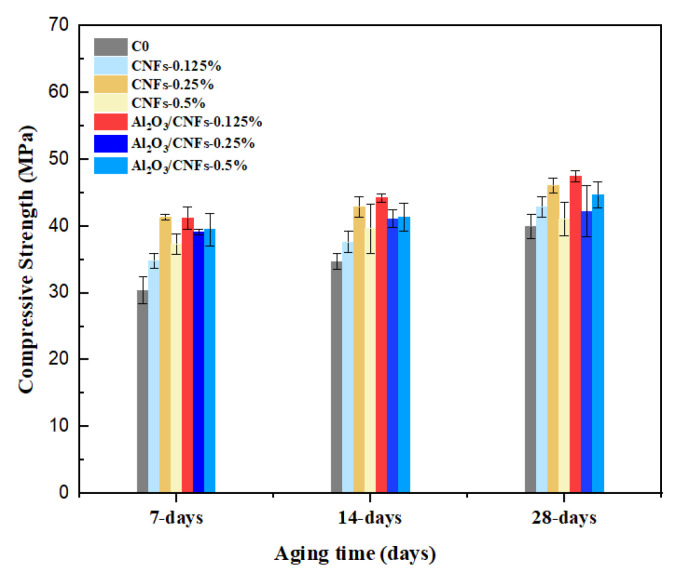
Compressive strength for a period of 7, 14, and 28 days.

The compressive strength results of cement mortars reinforced by CNFs and Al_2_O_3_/CNFs are presented in Figure 9. These results are displayed based on the nanomaterials’ weight percentage at the ages of 7, 14, and 28 days. These samples were compared with C0 at all ages. It was observed that with increasing curing age, all samples experienced an increase in their strength. The obtained compressive strength varies at all ages with different contents of CNFs and Al_2_O_3_/CNFs. The compressive strength for samples that contained CNFs at 0.125%, 0.25%, and 0.5% by weight of cement increased by about 15%, 36%, and 23%, respectively, at the age of 7 days. At the age of 14 days, the compressive strength of these composites increased by 8%, 24%, and 14%, respectively. When these samples reached a curing age of 28 days, the improvement in compressive strength was 6%, 15%, and 3%, respectively.

The improvement in the compressive strength with different dosages of CNFs was due to the combined effects of the crack bridging and microstructure densifying due to refining the porosity [48]. In addition, the surface-functionalized CNFs possess active surface sites such as -COOH, -OH, -C=O. These groups have an ability to improve the bond with Ca^2+^ and to accelerate cement hydration, improving the mechanical strength as a result [49]. Out of the CNFs samples, CNFs-0.25% showed the highest strength due to the structure compacting and refinement of porosity. The filling effect of the CNFs can be clearly observed from the SEM images (Figure 4b). There was a reduction in the compressive strength when the ratio of the CNFs increased to 0.5%. This could be due to the agglomeration of CNFs inside the cement mortar matrix. The agglomeration creates large cavities which lower the strength [18].

On the other hand, the composites that contained Al_2_O_3_/CNFs showed progressive improvement. Relative to C0, the addition of 0.125% of Al_2_O_3_/CNFs increased the strength by 36%, 27%, and 19% at the ages of 7, 14, and 28 days, respectively. In the case of Al_2_O_3_/CNFs-0.25, the strength exceeded the compressive strength of the control sample by about 29%, 19%, and 6% at the ages of 7, 14, and 28 days. When increasing the dosage of Al_2_O_3_/CNFs powder to 0.5%, the strength of the samples was increased by 30%, 19%, and 12%, respectively. The reason behind the good performance of the Al_2_O_3_/CNFs nanomaterials is the stronger interfacial bonding between the porous layer of the alumina on CNFs with cement matrix. Interestingly, the quantity of the CNFs in Al_2_O_3_/CNFs samples was reduced by 42% compared to CNF samples. The effect of the coating layer promoted the mechanical properties of the composites significantly. This layer possesses a highly reactive surface area that promotes the nucleation effect. The reactivity of the surface is defined as highly populated active sites that can efficiently adsorb and convert the calcium to calcium–silicate hydrate products. This layer may also contribute to producing extra hydration products, which further enhance the pore refinement and densify the cement matrix. Compressive strength enhancements obtained in this paper are compared to those available in the literature (Table 2).

### 3.6. Fluidity

The flow table diameter results are presented in Table 1. With different percentages of the nanomaterials and superplasticizer (SP), the mortar showed different fluidity performances. The control samples’ fluidity was only 133 mm. The fluidity diameters increased remarkably with the addition of CNFs-SP and Al_2_O_3_\CNFs-SP at various ratios. However, the fresh mortars reinforced with Al_2_O_3_/CNFs showed a noticeable reduction in flow spread compared with the mortars with different CNF ratios. This reduction in the workability was due to the large surface area that characterized the Al_2_O_3_-coated CNFs. The high surface area of the nanomaterial needs more water to wet its surface; this need can reduce the free water in the mixture [10]. Increasing the dosages of the SP is important to offset the reduction in the fluidity of mortars reinforced with Al_2_O_3_/CNFs at different ratios.

### 3.7. Freeze–Thaw Effects

The effect of the addition of the nanomaterials on the freeze–thaw performance of cement composites is evaluated in this section. The freeze–thaw performance in terms of physical and mechanical properties is described next.

#### 3.7.1. Compressive Strength

**Figure 10:**
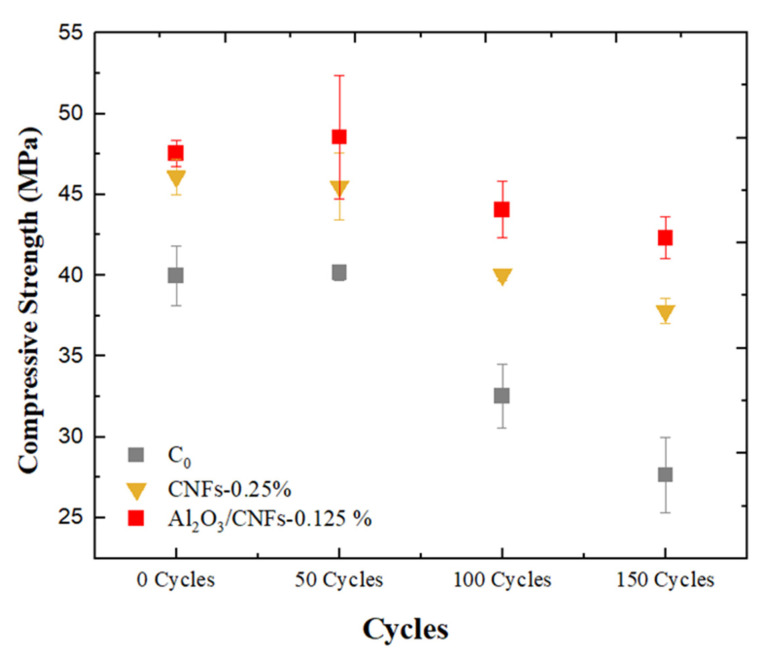
Compressive strength for C0, CNFs-0.125%, and Al_2_O_3_ /CNFs-0.125% composites after exposure to freezing–thawing cycles.

Figure 10 shows compressive strength versus the number of freeze–thaw cycles at 50, 100, and 150 cycles for C0, CNFs-0.25%, and Al_2_O_3_/CNFs-0.125% samples. Overall, samples with nanomaterials showed less compressive reduction when compared to C0 control samples. C0 displayed a decrease of 45% in compressive strength after 150 cycles, whereas specimens containing CNFs-0.25% showed a decrease of 22%. Interestingly, Al_2_O_3_/CNFs-0.125% had a significant enhancement in mortars’ frost resistance, showing the lowest compressive strength reduction of 14%. This performance was confirmed by the Barrett–Joyner–Halenda (BJH) analysis, as demonstrated in the section pore structure below. The BJH indicates the ability of Al_2_O_3_/CNFs to further refine the small and medium capillary pores, resulting in more compacted and durable composites.

#### 3.7.2. Mass Loss

**Figure 11:**
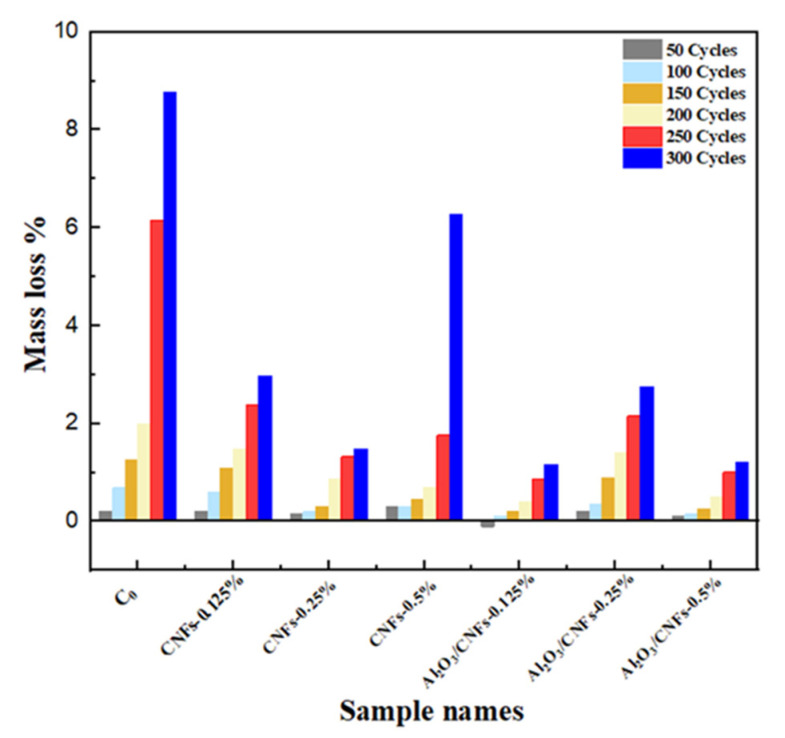
Mass loss ratios of mortar samples exposed to freezing–thawing cycles.

The weight changes were monitored during the freeze–thaw cycles, which were conducted after 50, 100, 150, 200, 250, and 300 cycles. As shown in Figure 11, the mass loss for all mixtures increased with increasing the number of freeze–thaw cycles. With the addition of nanomaterials, the mass loss in the samples was significantly reduced. In the case of the control sample C0, the average mass loss was about 9% after 300 cycles, while the addition of CNFs at different ratios always lowered mass loss, with the lowest being observed for the case of CNFs-0.25%. This may indicate that the addition of the functionalized CNFs worked to reduce porosity and increase the density of the CNF/cement composites. However, the mass loss increased when CNFs-0.5% was embedded in cement mortars. This was expected as these composites showed lower strength due to the large pores that were created in the matrix because of the agglomeration of the CNFs bundles.

**Figure 12:**
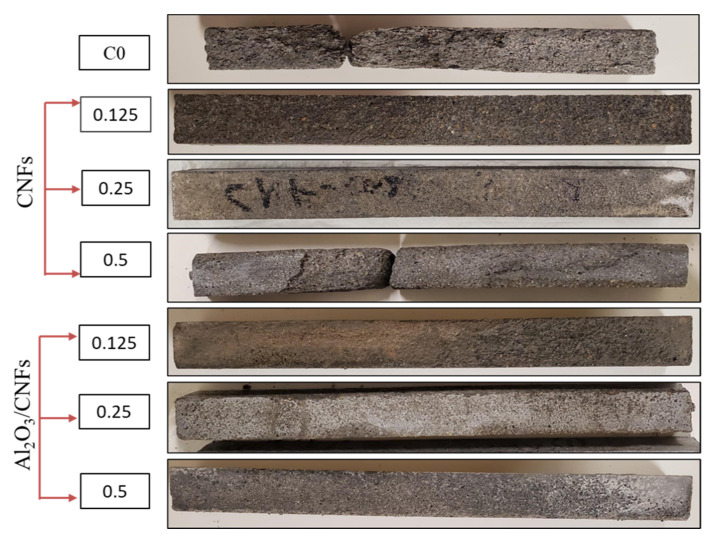
Physical appearance of the samples after 300 cycles.

Following the same trend, the addition of Al_2_O_3_/CNFs showed a remarkable performance in resisting frost. Overall, the addition of different ratios of Al_2_O_3_/CNFs enhanced the frost resistance. The addition of Al_2_O_3_/CNFs-0.125% and Al_2_O_3_/CNFs-0.5% lead to a significantly lower mass loss after 300 cycles by only 1%. This improvement is explained by the decreasing porosity resulting from modifying the internal voids. This might be due to the refinement of the pores’ structure by producing additional hydrated gels, which prevent intrusion of water molecules into the mixture’s microstructure. Furthermore, this refinement could be due to the reactivity of the porous Al_2_O_3_ layer, which accelerates the hydration process, forming more well-distributed C-S-H hydrated gels in the matrix. Fewer CH crystals and more amorphous C-S-H in Al_2_O_3_/CNFs-0.125%, as shown in SEM, XRD, and TGA/DTA, is a testament of this speculation. The small-size voids could minimize the water settling time and subsequently retard the ice volume enlargement. This is expected since a small-size void would hold a high internal pressure and surface tension based on physics principles. The physical appearance of all samples after 300 cycles is presented in Figure 12. Clearly, the addition of the nanomaterials with good dispersion in the mortar matrix enhanced the frost resistance. The high frost resistance was observed in Al_2_O_3_/CNFs composites.

#### 3.7.3. Pore Structure

**Figure 13:**
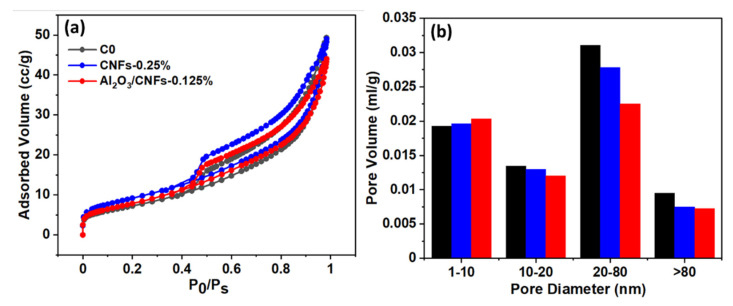
(**a**) BET isotherm analysis, (**b**) pore volume distribution.

The cement mortar powder was grained and filtered by a 300 µm sieve, and then it was dried and vacuumed at 200 °C for 2 h during the degassing process. The pore volume of the samples was calculated based on the BJH method [55]. The overall BJH pore volume was also found to be 0.07805, 0.06783, and 0.06278 mL/g for C0, CNFs-0.25%, and Al_2_O_3_/CNFs-0.125%, respectively. Figure 13a shows a type IV isotherm hysteresis for adsorption–desorption [56]. Figure 13b shows the pore measurement statistics for the three samples. The pore diameter statistic is divided into four regions. It can be seen that the Al_2_O_3_/CNFs-0.125% had pores with a greater volume in the range of 1–10 nm, which are classified as gel pores [57]. The gel pores are related to the formation of C-S-H, which is often produced by the hydration reaction of C_3_S and C_2_S. In other ranges, where the capillary pores are present, the Al_2_O_3_/CNFs-0.125% pore volume was significantly reduced, indicating the refinement was enhanced. The pore refinement was also induced when CNFs were incorporated; however, Al_2_O_3_/CNFs-0.125% further reduced the pore volume, which may be due to the higher C-S-H content, as described in TGA and XRD results. It may also be due to the pozzolanic reaction offered by the Al_2_O_3_ nanocoating, which helps produce extra C-A-H. Recently, a study has demonstrated the effect of different aluminum oxide phases (Al(OH)_3_, γ-Al_2_O_3_, α-Al_2_O_3_) on the calcium aluminate cement hydration [58]. In another study, it was found that Al(OH)_3_ significantly accelerates hydration due to the high surface area and the ability to serve as a nucleation center for C-A-H moieties [59]. This is in line with the results obtained in this study, as aluminum oxide was in the boehmite phase, as confirmed by the XRD shown in this reference [35].

### 3.8. Drying Shrinkage

**Figure 14:**
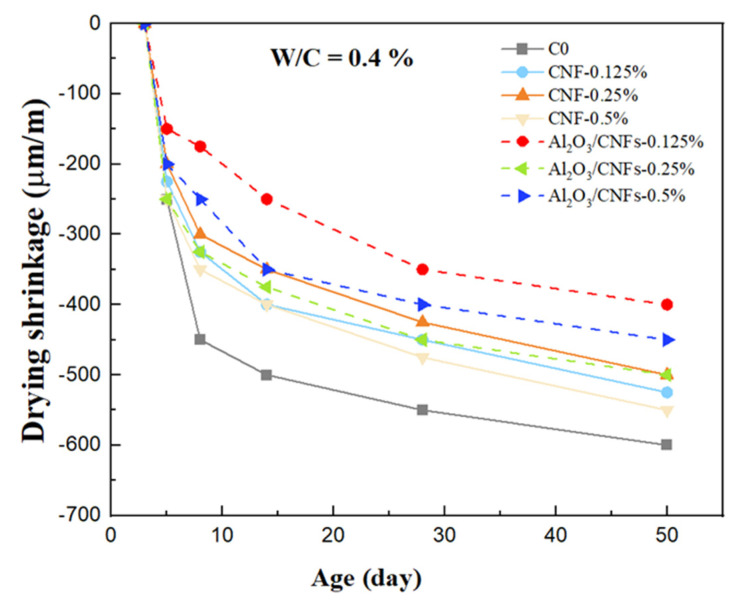
Drying shrinkage data for all the samples.

The effect of the addition of the nanomaterials on the drying shrinkage was studied at a water/cement ratio of 0.40. The results, as shown in Figure 14, indicate that the cement mortars with CNFs and Al_2_O_3_-coated CNFs had lower shrinkage compared to the C0 control mortar at early and late ages. In comparison with C0, the average early drying shrinkage of composites that were reinforced by CNFs at ratios of 0.125%, 0.25%, and 0.5% showed noticeable decreases of about 19%, 33%, and 22%, respectively, at the age of 7 days, while after 56 days, the reduction was about 16%, 20%, and 14%. In addition, the results show a remarkable reduction in the drying shrinkage for the composites that contained Al_2_O_3_/CNFs at various ratios. It is clear that the addition of the Al_2_O_3_/CNFs-0.125% significantly reduced the shrinkage relative to other samples, whether in the early or late ages. At age of 7 days and 56 days, Al_2_O_3_/CNFs-0.125% reduced the shrinkage by 62% and 47%, respectively, compared to the control C0 mortar. The Al_2_O_3_/CNFs-0.25% mortar showed 36% and 27% shrinkage reduction during the same time periods. On the other hand, the composites that contained 0.5% of Al_2_O_3_/CNFs exhibited low shrinkage by 30% and 17% at the age of 7 and 56 days, respectively.

Drying shrinkage of cementitious materials is due to capillary surface tension because of the meniscus formation in the capillary pores [60]. The incorporation of both CNFs and Al_2_O_3_/CNFs in the mortar matrix reduced the total overall porosity. In addition, increasing the stiffness due to the microcrack bridging effect of these nanomaterials can be an effective way to reduce the shrinkage deformation. Furthermore, the Al_2_O_3_ porous layer worked as a reactive site to increase the nucleation, which worked to densify and to compact the microstructure of the matrix. The refinement in capillary pores served as an obstacle impeding the water loss from the cement skeleton [60,61]. The mitigation of the drying shrinkage of the Al_2_O_3_/CNFs composites could be due to the high hydrophilicity of these nanomaterials. The Al_2_O_3_ nanocoating is in a boehmite phase [35], and thus has a strong ability to absorb and retain water as long as it is in equilibrium with surrounding cement compounds. When the shrinkage takes place, the water is released for the gel to achieve the internal hydration equilibrium [62].

## 4. Limitations of the Study

This study investigated the effects of using Al_2_O_3_/CNFs on compressive strength, durability under freeze–thaw cycles, and drying shrinkage of cement mortars. Although the results have proven the viability of the approach, the study had many constraints. Firstly, the stability of the Al_2_O_3_ nanolayer in high concentrations of the superplasticizer, which is an acidic agent, is unknown so far. Secondly, the effect of the alumina-coated carbon nanofibers on drying shrinkage with different water-to-cement ratios and various temperatures is not included in this study. Finally, the degree of pores’ refinements due to Al_2_O_3_/CNFs incorporation has not been measured by a direct technique such as water absorption or mercury intrusion porosimeter.

## 5. Conclusions

Based on the results presented in this paper, the following conclusions were drawn:

1. The TEM images revealed a well-coated layer on the CNF surfaces.
2. The SEM results show the nanomaterials were well-distributed in the mortar microstructure.
3. The SEM images indicate the formation of the hydration products gels such as CH, C-S-H, and ettringite. In the case of the Al_2_O_3_/CNFs-mortar composite, most of the hydrated products were well-distributed C-S-H and were identified from their amorphous appearance, while CNFs-mortar composites showed mostly ettringite and hexagonal CH.
4. With increasing curing age, all samples experienced an increase in their compressive strength. The compressive strength for samples that contained Al_2_O_3_/CNFs demonstrated progressive improvements compared to other samples evaluated in this research. Relative to control specimens, the addition of Al_2_O_3_/CNFs-0.125% increased the strength by 36%, 27%, and 19% at the age of 7, 14, and 28 days, respectively. The alumina porous layer improved the binding between the mortar matrix and the CNFs’ surfaces and altered the hydration process.
5. TGA-DTA tests showed all CNFs and Al_2_O_3_/CNF composites exhibited higher degrees of hydration than the control sample. Decreasing calcium hydroxide was observed at an age of 28 days with the addition of the nanomaterials, with a significant effect for the case of coated CNFs. This could be due to the nucleation effect, which produced a good distribution of the gel hydration of C-S-H inside the mortar matrix, which restricts the growth of the CH. Additionally, this may be due to the pozzolanic effect of the Al_2_O_3_ layer. There might be some minor overlapping in the signal from CNFs burning and CH diminishing.
6. The XRD measurement at the age of 28 days focused on the intensity of two cementitious moieties: the C_2_S and C_3_S to be consumed in the hydration reaction. Al_2_O_3_/CNFs-0.125% showed the lowest intensity of these two compounds, which indicates that they increased the dissolution of C_3_S and C_2_S, and thus more C-S-H was produced. In addition, XRD data focused on the intensity of CH, as it showed lower intensity in the case of CNFs-0.25% and Al_2_O_3_/CNFs 0.125% composites. These results are in good agreement with TGA and EDS analyses findings.
7. The addition of Al_2_O_3_/CNFs-0.125% and Al_2_O_3_/CNFs-0.5% showed a significant low mass loss of about 1% after 300 freeze–thaw cycles, while the reference sample and CNFs-0.25 showed a mass loss of 9% and 3%, respectively.
8. The compressive strength degradation was the lowest in the case of the Al_2_O_3_/CNFs-0.125% sample after 150 freeze–thaw cycles at about 14%, while the control and CNFs-0.25% samples showed a degradation of about 45 and 22%, respectively. Such results were attributed to the highly dense microstructure, which was later confirmed by the BET measurements. The BET and BJH tests exhibited low gas adsorption and more refinement in the capillary pores.
9. Incorporation of the optimum amounts of CNFs (0.25%) and Al_2_O_3_/CNFs (0.125%) led to a decrease in the drying shrinkage due to increasing the stiffness and density of these composites. In addition, the hydrophilicity of the alumina layer explained the significant drying shrinkage mitigation.

## Figures and Tables

**Table 1:** Ingredient proportions for cement mortar preparation.

Sample Designation	Water-to-Cement Ratio (w/c)	Sand-to-Cement Ratio (s/c)	Mix Proportion (% Weight)			Fluidity (mm)	CNF Content (%wt) *
			CNFs	Al_2_O_3_/CNFs	Superplasticizer		
C0	0.35	2	-	-	0.1	133	-
CNF-0.125	0.35	2	0.125	-	0.1	156	100
CNF-0.25	0.35	2	0.25	-	0.2	158	100
CNF-0.5	0.35	2	0.5	-	0.4	164	100
Al_2_O_3_/CNF-0.125	0.35	2	-	0.125	0.1	151	58
Al_2_O_3_/CNF-0.25	0.35	2	-	0.25	0.2	150	58
Al_2_O_3_/CNF-0.5	0.35	2	-	0.5	0.4	161	58

* Taken from TGA data.

**Table 2:** Compressive strength comparison obtained in this paper with existing research.

Property	Enhancement in Properties	Material Concentration	Reference
Compressive strength	36%	0.125% Al_2_O_3_/CNFs	This study
	19%	0.5 wt.% MWCNTs	Li et al. [50]
	22%	0.1 wt.% MWCNTs	Bharj et al. [51]
	42.7%	0.16 wt.% CNFs	Gao et al. [52]
	25.4%	0.5 wt.% SiO_2_/MWCNTs	Shaojie et al. [53]
	20%	0.125 wt.% MWCNTs/NSs	Pawel et al. [31]
	40%	0.5 wt.% CNT/TNT	Liu et al. [54]

## Data Availability

The data presented in this study are available on request from the corresponding author. The data are not publicly available due to copyright.

## References

[1] Ateş A. (2016). Mechanical properties of sandy soils reinforced with cement and randomly distributed glass fibers (GRC). Compos. Part B Eng..

[2] Balaguru P.N., Shah S.P. (1992). Fiber-Reinforced Cement Composites.

[3] Bentur A., Mindess S. (2006). Fibre Reinforced Cementitious Composites.

[4] Banthia N., Sappakittipakorn M. (2007). Toughness enhancement in steel fiber reinforced concrete through fiber hybridization. Cem. Concr. Res..

[5] Pigeon M. (2014). Durability of Concrete in Cold Climates.

[6] del Carmen Camacho M., Galao O., Baeza F.J., Zornoza E., Garcés P. (2014). Mechanical properties and durability of CNT cement composites. Mater. Des..

[7] Camacho-Ballesta C., Galao Ó., Baeza F.J., Zornoza E., Garcés P. (2019). Durability and Mechanical Properties of CNT Cement Composites.

[8] Nivethitha D., Srividhya S., Dharmar S. (2016). Review on mechanical properties of cement mortar enhanced with nanoparticles. Int. J. Sci. Res. Dev. News R D News.

[9] Wang T., Xu J., Meng B., Peng G. (2020). Experimental study on the effect of carbon nanofiber content on the durability of concrete. Constr. Build. Mater..

[10] Chuah S., Pan Z., Sanjayan J.G., Wang C.M., Duan W.H. (2014). Nano reinforced cement and concrete composites and new perspective from graphene oxide. Constr. Build. Mater..

[11] Wang F., Kong X., Jiang L., Wang D. (2020). The acceleration mechanism of nano-C-S-H particles on OPC hydration. Constr. Build. Mater..

[12] Ebrahimi K., Daiezadeh M.J., Zakertabrizi M., Zahmatkesh F., Korayem A.H. (2018). A review of the impact of micro- and nanoparticles on freeze-thaw durability of hardened concrete: Mechanism perspective. Constr. Build. Mater..

[13] Kumar S., Kolay P., Malla S., Mishra S. (2015). Effect of Multiwalled Carbon Nanotube in Cement Composite on Mechanical Strength and Freeze-Thaw Susceptibility. Adv. Civ. Eng. Mater..

[14] Lee H., Jeong S., Cho S., Chung W. (2020). Enhanced bonding behavior of multi-walled carbon nanotube cement composites and reinforcing bars. Compos. Struct..

[15] Al-Rub R.K.A., Ashour A.I., Tyson B.M. (2012). On the aspect ratio effect of multi-walled carbon nanotube reinforcements on the mechanical properties of cementitious nanocomposites. Constr. Build. Mater..

[16] Cwirzen A., Habermehl-Cwirzen K. (2013). The effect of carbon nano-and microfibers on strength and residual cumulative strain of mortars subjected to freeze-thaw cycles. J. Adv. Concr. Technol..

[17] Metaxa Z.S., Konsta-Gdoutos M.S., Shah S.P. (2010). Carbon Nanofiber–Reinforced Cement-Based Materials. Transp. Res. Rec. J. Transp. Res. Board.

[18] Shi T., Li Z., Guo J., Gong H., Gu C. (2019). Research progress on CNTs/CNFs-modified cement-based composites—A review. Constr. Build. Mater..

[19] Givi A.N., Rashid S.A., Aziz F.N.A., Salleh M.A.M. (2010). Experimental investigation of the size effects of SiO2 nano-particles on the mechanical properties of binary blended concrete. Compos. Part B Eng..

[20] Reches Y. (2018). Nanoparticles as concrete additives: Review and perspectives. Constr. Build. Mater..

[21] Behfarnia K., Salemi N. (2013). The effects of nano-silica and nano-alumina on frost resistance of normal concrete. Constr. Build. Mater..

[22] El-Gamal S.M.A., Abo-El-Enein S.A., El-Hosiny F.I., Amin M.S., Ramadan M. (2018). Thermal resistance, microstructure and mechanical properties of type I Portland cement pastes containing low-cost nanoparticles. J. Therm. Anal..

[23] Nazari A., Riahi S., Riahi S., Shamekhi S.F., Khademno A. (2010). Influence of Al_2_O_3_ nanoparticles on the compressive strength and workability of blended concrete. J. Am. Sci..

[24] Muzenski S., Flores-Vivian I., Sobolev K. (2019). Ultra-high strength cement-based composites designed with aluminum oxide nano-fibers. Constr. Build. Mater..

[25] Fu X., Lu W., Chung D.D.L. (1998). Ozone treatment of carbon fiber for reinforcing cement. Carbon.

[26] Wang J., Cheng Y., Yuan L., Xu D., Du P., Hou P., Zhou Z., Cheng X., Liu S., Wang Y. (2020). Effect of nano-silica on chemical and volume shrinkage of cement-based composites. Constr. Build. Mater..

[27] Abdel-Gawwad H.A., Mohammed M.S., Alomayri T. (2019). Single and dual effects of magnesia and alumina nano-particles on strength and drying shrinkage of alkali activated slag. Constr. Build. Mater..

[28] Sanchez F., Ince C. (2009). Microstructure and macroscopic properties of hybrid carbon nanofiber/silica fume cement composites. Compos. Sci. Technol..

[29] Yazdanbakhsh A., Grasley Z. (2014). Utilization of Silica Fume to Stabilize the Dispersion of Carbon Nanofilaments in Cement Paste. J. Mater. Civ. Eng..

[30] Stynoski P., Mondal P., Marsh C. (2015). Effects of silica additives on fracture properties of carbon nanotube and carbon fiber reinforced Portland cement mortar. Cem. Concr. Compos..

[31] Sikora P., Elrahman M.A., Chung S.-Y., Cendrowski K., Mijowska E., Stephan D. (2019). Mechanical and microstructural properties of cement pastes containing carbon nanotubes and carbon nanotube-silica core-shell structures, exposed to elevated temperature. Cem. Concr. Compos..

[32] Garg M., Das C.S., Gupta R. (2020). Use of silica particles to improve dispersion of -COOH CNTs/carbon fibers to produce HyFRCC. Constr. Build. Mater..

[33] Kim H.-K., Nam I., Lee H. (2014). Enhanced effect of carbon nanotube on mechanical and electrical properties of cement composites by incorporation of silica fume. Compos. Struct..

[34] Dong S., Wang D., Ashour A., Han B., Ou J. (2021). Nickel plated carbon nanotubes reinforcing concrete composites: From nano/micro structures to macro mechanical properties. Compos. Part A: Appl. Sci. Manuf..

[35] Jasim A.M., He X., White T.A., Xing Y. (2020). Nano-layer deposition of metal oxides via a condensed water film. Commun. Mater..

[36] (2013). Standard Test Method for Flow of Hydraulic Cement Mortar. ASTM C1437-13.

[37] (1999). Standard Test Method for Compressive Strength of Hydraulic Cement Mortars (Using 2-in. or (50-mm) Cube Specimens). ASTM C109/C109M.

[38] (2008). Standard Test Method for Resistance of Concrete to Rapid Freezing and Thawing. ASTM C666–03.

[39] (2018). Standard Test Method for Drying Shrinkage of Mortar Containing Hydraulic Cement. ASTM C596-18.

[40] (2011). Standard Practice for Use of Apparatus for the Determination of Length Change of Hardened Cement Paste, Mortar, and Concrete. ASTM C490-07.

[41] da Silva Andrade D., da Silva Rêgo J.H., Morais P.C., de Mendonça Lopes A.N., Rojas M.F. (2019). Investigation of C-S-H in ternary cement pastes containing nanosilica and highly-reactive supplementary cementitious materials (SCMs): Microstructure and strength. Constr. Build. Mater..

[42] Al Qader H.J.N. (2021). Enhancing the Mechanical and Durability Properties of Cement Mortars by using Alumina Nanocoating on Carbon Nanofibers. Master Thesis.

[43] Kumar S., Rath T., Mahaling R.N., Das C.K. (2007). Processing and characterization of carbon nanofiber/syndiotactic polystyrene composites in the absence and presence of liquid crystalline polymer. Compos. Part A Appl. Sci. Manuf..

[44] Deboucha W., Leklou N., Khelidj A., Oudjit M.N. (2017). Hydration development of mineral additives blended cement using thermogravimetric analysis (TGA): Methodology of calculating the degree of hydration. Constr. Build. Mater..

[45] Makar J.M., Chan G.W. (2009). Growth of Cement Hydration Products on Single-Walled Carbon Nanotubes. J. Am. Ceram. Soc..

[46] Tafesse M., Kim H.-K. (2019). The role of carbon nanotube on hydration kinetics and shrinkage of cement composite. Compos. Part B: Eng..

[47] Mahinroosta M., Allahverdi A. (2019). A Scoping Review on Integrating Inorganic Nanomaterials into Cement Composites. Adv. Civ. Eng. Mater..

[48] Fehervari A., MacLeod A.J.N., Garcez E.O., Aldridge L., Gates W.P., Yang Y., Collins F. (2020). On the mechanisms for improved strengths of carbon nanofiber-enriched mortars. Cem. Concr. Res..

[49] Peyvandi A., Soroushian P., Abdol N., Balachandra A.M. (2013). Surface-modified graphite nanomaterials for improved reinforcement efficiency in cementitious paste. Carbon.

[50] Li G.Y., Wang P.M., Zhao X. (2005). Mechanical behavior and microstructure of cement composites incorporating surface-treated multi-walled carbon nanotubes. Carbon.

[51] Bharj J. (2015). Experimental study on compressive strength of cement-CNT composite paste. Indian J. Pure Appl. Phys..

[52] Gao D., Sturm M., Mo Y. (2009). Electrical resistance of carbon-nanofiber concrete. Smart Mater. Struct..

[53] Li S., Zhang Y., Lin S., Yan J., Du S. (2021). Effects of nano-SiO2 coated multi-walled carbon nanotubes on mechanical properties of cement-based composites. Constr. Build. Mater..

[54] Liu J., Suh H., Jee H., Xu J., Nezhad E.Z., Choi C.-S., Bae S. (2021). Synergistic effect of carbon nanotube/TiO_2_ nanotube multi-scale reinforcement on the mechanical properties and hydration process of portland cement paste. Constr. Build. Mater..

[55] Shafaei D., Yang S., Berlouis L., Minto J. (2020). Multiscale pore structure analysis of nano titanium dioxide cement mortar composite. Mater. Today Commun..

[56] Vardharajula S., Ali S.Z., Tiwari P.M., Eroğlu E., Vig K., Dennis V.A., Singh S.R. (2012). Functionalized carbon nanotubes: Biomedical applications. Int. J. Nanomed..

[57] Meng W., Khayat K.H. (2018). Effect of graphite nanoplatelets and carbon nanofibers on rheology, hydration, shrinkage, mechanical properties, and microstructure of UHPC. Cem. Concr. Res..

[58] Engbert A., Plank J. (2021). Impact of sand and filler materials on the hydration behavior of calcium aluminate cement. J. Am. Ceram. Soc..

[59] Reches Y., Thomson K., Helbing M., Kosson D.S., Sanchez F. (2018). Agglomeration and reactivity of nanoparticles of SiO_2_, TiO_2_, Al_2_O_3_, Fe_2_O_3_, and clays in cement pastes and effects on compressive strength at ambient and elevated temperatures. Constr. Build. Mater..

[60] Young J., Bazant Z.P. (1988). Physical mechanisms and their mathematical descriptions. Mathematical Modelling of Creep and Shrinkage of Concrete.

[61] Hawreen A., Bogas J., Dias A.P.S. (2018). On the mechanical and shrinkage behavior of cement mortars reinforced with carbon nanotubes. Constr. Build. Mater..

[62] Liu X., Fang T., Zuo J. (2019). Effect of Nano-Materials on Autogenous Shrinkage Properties of Cement Based Materials. Symmetry.

